# Assessment of DNA Methylation and Oxidative Changes in the Heart and Brain of Rats Receiving a High-Fat Diet Supplemented with Various Forms of Chromium

**DOI:** 10.3390/ani10091470

**Published:** 2020-08-21

**Authors:** Wojciech Dworzański, Ewelina Cholewińska, Bartosz Fotschki, Jerzy Juśkiewicz, Piotr Listos, Katarzyna Ognik

**Affiliations:** 1 Chair and Department of Human Anatomy, Medical University of Lublin, Jaczewskiego 4, 20-090 Lublin, Poland; wojciech.dworzanski@umlub.pl; 2 Department of Biochemistry and Toxicology, Faculty of Animal Sciences and Bioeconomy, University of Life Sciences in Lublin, Akademicka 13, 20-950 Lublin, Poland; kasiaognik@poczta.fm; 3 Animal Reproduction and Food Research, Polish Academy of Sciences, Tuwima 10, 10–748 Olsztyn, Poland; b.fotschki@pan.olsztyn.pl (B.F.); j.juskiewicz@pan.olsztyn.pl (J.J.); 4 Sub-Department of Pathomorphology and Forensic Medicine, Faculty of Veterinary Medicine, University of Life Sciences in Lublin, Głęboka 30, 20-612 Lublin, Poland; piotr.listos@up.lublin.pl

**Keywords:** oxidative stress, epigenetic changes, DNA repair enzymes, chromium picolinate, chromium (III)-methionine, chromium nanoparticles

## Abstract

**Simple Summary:**

Obesity is one of the most prevalent diseases of civilization in the 21st century. This may be due to an increase in the consumption of high-fat diets. For the treatment of obesity, various supplements with chromium (III) are used. Chromium has the ability to regulate carbohydrate and lipid metabolism, which may result in weight loss. Our studies compared the effects of a high-fat diet supplemented with three different forms of chromium-chromium (III) picolinate, chromium (III) -methionine, and nano-sized chromium on oxidative damage of the DNA, proteins, and lipids in the heart and brain of rats. The conducted study showed that the use of a high-fat diet results in oxidation of the DNA, proteins, and lipids in the brain and heart, and the addition of chromium additionally intensifies these processes, especially when used in the form of nanoparticles. Therefore, the results of these studies suggest that one should be careful when using chromium supplementation to counteract obesity, because it may be associated with the risk of deterioration of the functioning of the heart and brain.

**Abstract:**

The aim of the study was to determine how feeding rats a high-fat diet supplemented with various forms of chromium affects DNA methylation and oxidation reactions as well as the histology of heart and brain tissue. The rats received standard diet or high-fat diet and chromium at 0.3 mg/kg body weight (BW) in form of chromium (III) picolinate, chromium (III)-methionine, or nano-sized chromium. The content of malondialdehyde (MDA), protein carbonyl (PC), and 8-hydroxydeoxyguanosine (8-OHDG), the level of global DNA methylation and the activity of selected DNA repair enzymes were determined in the blood. In the brain and heart, the content of MDA, PC, 8-OHDG, and levels of global DNA methylation were determined. The brain was subjected to histological examination. The use of a high-fat diet was found to intensify epigenetic changes and oxidation reactions in the heart and brain. It was concluded that epigenetic changes and oxidation of lipids, proteins, and DNA in the heart and brain of rats resulting from the use of a high-fat diet cannot be limited by supplementing the diet with chromium. It was established that the use of chromium to supplement a high-fat diet intensifies the negative epigenetic and oxidative changes in the heart and brain, especially in the case of chromium nanoparticles.

## 1. Introduction

Obesity is one of the most prevalent diseases of civilization in the 21st century. It can be influenced by genetic, epigenetic, socio-economic, biological, and behavioural factors [1,2]. However, a high-fat diet seems to be of particular importance in the pathophysiology of obesity. Obesity, especially visceral obesity, is the main component of metabolic syndrome, which is a combination of related factors increasing the risk of type 2 diabetes and cardiovascular disease, including hypertension, stroke, coronary artery disease, heart failure, and cardiomyopathy [3,4]. Insulin resistance, hyperinsulinaemia, and hyperleptinaemia are also commonly observed in obese people [3]. There are also reports indicating that excessive fat consumption and obesity adversely affect the nervous system, increasing the risk of dementia and Alzheimer’s disease [5,6]. Impaired functioning of the body due to a high-fat diet is believed to result from the excessive production of reactive oxygen species (ROS) that accompany it [7,8].

Chromium (III) (Cr) is a key microelement involved in the metabolism of carbohydrates, proteins, and fats in humans and animals [9]. There are also reports indicating that chromium (III) is involved in the metabolism of nucleic acid [10] and favourably stimulates the immune response and disease resistance [11,12]. Cr via stimulation of insulin activity may favorably enhance the processes of β-amyloid removal in the central nervous system (CNS). Furthermore, insulin present in the CNS participates in the body’s energy regulation, including appetite control [13]. Due to these properties of chromium, particularly its ability to regulate carbohydrate-lipid metabolism and reduce body weight, it is popularly used as a factor supporting the treatment of type 2 diabetes and as a component of supplements used in slimming treatments [14,15].

Currently, the most popular form of chromium used in dietary supplements is picolinate, an organic compound of trivalent Cr [15,16]. The available literature indicates that its bioavailability is higher than that of its inorganic counterparts, such as CrCl_3_ [15]. The use of chromium picolinate has many benefits, including weight reduction and increased muscle mass [17]. However, due to the relatively low bioavailability of chromium picolinate, other forms of this element are sought that could be better utilized by the body. For this reason, researchers are increasingly interested in complexes of Cr with amino acids, such as methionine or glycine [18,19] as well as inorganic chromium nanoparticles [20]. It is believed that the lack of electric charge and the small size of chromium nanoparticles can modify their properties in a living organism and improve bioavailability relative to their standard counterparts [21].

In this study, we postulated that a high-fat diet given to rats intensifies epigenetic changes and oxidation of lipids, proteins, and DNA in heart and brain tissues, but that there is a form of chromium that limits negative epigenetic and oxidative changes. The aim of the study was to determine how feeding rats a high-fat diet supplemented with various forms of chromium affects DNA methylation and oxidation reactions as well as the histology of heart and brain tissue.

## 2. Materials and Methods

### 2.1. Forms of Chrome Used in the Experiment

Chromium picolinate (Cr-Pic; purity >980 g/kg) was purchased from Sigma-Aldrich Sp. z o.o. (Poznan, Poland). Chromium-methionine complex (Cr-Met) was purchased from Innobio Co., Ltd. (Siheung, Korea). Chromium nanopowder (Cr-NPs) with 99.9% purity, size 60~80 nm, spherical shape, specific surface area 6–8 m^2^/g, bulk density 0.15 g/cm^3^, and true density 8.9 g/cm^3^ was produced and purchased from SkySpring Nanomaterials (Houston, TX, USA).

### 2.2. Animals and Diets

The experiment was conducted on 56 male outbred Wistar rats (*Rattus norvegicus*. Cmdb:WI). The animals were used in compliance with the European Guidelines for the Care and Use of Laboratory Animals [22]. The experimental protocol was approved by the Local Animal Care and Use Committee (Approval No. 04/2019; Olsztyn. Poland). All efforts were made to minimize the suffering of the experimental animals. At the start of the experiment, rats aged 5 weeks and weighing 131 ± 4.33 g were randomly assigned to one of eight groups of seven rats each. The animals were kept individually in metabolic cages under a stable temperature (21–22 °C), a 12/12 h light/dark cycle, and a ventilation rate of 20 air changes per hour. For eight weeks, the rats had free access to tap water and semi-purified diets, which were prepared and then stored at 4 °C in hermetic containers until the end of the experiment (Table 1). The diets were modifications of a casein diet for laboratory rodents recommended by the American Institute of Nutrition [23]. Two types of diet were used: a standard diet (diet S) containing 8% rapeseed oil and 5% cellulose as sources of fat and dietary fibre, and a high-fat, low-fibre diet (diet F), which was a modification of diet S with 17% lard added in place of corn starch and cellulose content reduced to 3%. All diets were balanced for content of dietary protein from a casein preparation (20% of diet; Lacpol Co., Murowana Goslina, Poland) and DL-methionine (0.3% of diet) whereas the F diets excelled the S ones by 23% in caloric density due to the enhanced fat content (25 vs. 8% of diet, respectively). The various chromium sources prepared at a laboratory scale were added to the S and F diets at the same dosage, for a two-factorial experimental design (see description of statistical analyses). The amount of chromium administered to each rat was 0.3 mg/kg BW, selected according to the EFSA NDA Panel [24]. The sources of dietary Cr were chromium (III) picolinate (Cr-Pic), Chromium (III)-methionine (Cr-Met), and nano-sized chromium (Cr-NPs). For the safety of the operator preparing the experimental diets with the Cr-NP preparation, all Cr sources (to maintain comparable conditions) were added to the diet as an emulsion together with dietary rapeseed oil rather than in the mineral mixture.

### 2.3. Sample Collection and Analyses

At the end of the experiment, the rats were fasted for 12 h and anaesthetized *i.p.* with ketamine and xylazine (K. 100/kg BW; X. 10 mg/kg BW) according to the recommendations for anaesthesia and euthanasia of experimental animals. After laparotomy, blood samples were taken from the caudal vena cava into heparinized tubes, and finally the rats were euthanized by cervical dislocation. Then the heart and brain were dissected. The blood plasma was prepared by solidification and low-speed centrifugation (350× *g*. 10 min. 4 °C). Plasma samples were kept frozen at −70 °C until assayed.

### 2.4. Ex Vivo Analysis

#### 2.4.1. Oxidative Status in the Plasma

As a marker of oxidative stress, the concentration of the lipid oxidation indicator malondialdehyde (MDA) was determined in the blood using kits produced by Cell Biolabs, Inc., San Diego, CA, USA. OxiSelect diagnostic kits (Cell Biolabs, Inc., San Diego, CA, USA) were used to determine protein carbonyl (PC) derivatives as an indicator of oxidation of amino acid residues. The concentration of beta-amyloid was determined using a diagnostic kit from Cell Biolabs, Inc., San Diego, CA, USA. The concentration of 8-hydroxydeoxyguanosine (8-OHdG) was determined as a marker of oxidation of DNA bases. DNA was isolated from the blood using kits by QIAGEN. The level of epigenetic changes in the blood was determined by analysing global DNA methylation (methylome) using diagnostic kits by Sigma Aldrich. Activity of the enzymes superoxide dismutase (SOD) and glutathione peroxidase (GPx) in the blood of the rats was determined by spectrometry using Ransel and Ransod diagnostic kits manufactured by Randox (Poland). A diagnostic kit manufactured by Oxis International, Inc., Portland, USA, was used to determine catalase activity (CAT).

#### 2.4.2. Oxidative Status in the Brain and Heart

As a marker of oxidative stress in the brain and heart, the lipid oxidation indicator malondialdehyde (MDA) was determined using kits produced by Cell Biolabs, Inc., San Diego, CA, USA. OxiSelect diagnostic kits (Cell Biolabs, Inc., San Diego, CA, USA) were used to determine protein carbonyl (PC) derivatives as an indicator of oxidation of amino acid residues. The concentration of 8-hydroxydeoxyguanosine (8-OHdG) was determined as a marker of oxidation of DNA bases. DNA was isolated from the heart and brain using kits manufactured by QIAGEN as per the manufacturer’s instructions. The purity of DNA preparations was assessed by A260/A280 ratio. Extracted DNA probes were dissolved in sterile water at 2 µg/µL. Then, DNA samples were incubated at 95 °C for 5 min and rapidly chilling on ice for conversion to single-stranded DNA. Next, DNA samples were digested with nuclease P1 (5 units previously reconstituted in the manufacturer’s recommended buffer) by incubating at 37 °C for 2 h followed by treatment with alkaline phosphatase (AP) (5 units, previously reconstituted in the manufacturer’s recommended buffer) plus sufficient Tris buffer to a final concentration of 100 mM Tris, pH 7.5, and incubate at 37 °C for 1 h. The resulting reaction mixture was centrifuged at 6000× *g* for 5 min and the supernatant was collected for use in the ELISA. 8-OHdG levels were measured by using commercial measurement enzyme-linked immunosorbent assay (ELISA) kit (Cell Biolabs, Inc., San Diego, CA, USA). Absorbances were measured at 450 nm via ELISA reader. The level of epigenetic changes in the brain and heart was determined by analysing global DNA methylation (methylome) using diagnostic kits by Sigma Aldrich.

#### 2.4.3. DNA Repair Enzymes in the Blood

The activity of apurinic/apyrimidinic endonuclease 1 (APE-1), DNA-3-methyladenine glycosylase (ANPG) and thymine DNA glycosylase (TDG) was determined in the blood using OxiSelect diagnostic kits (Cell Biolabs, Inc., San Diego, CA, USA). The activity of low-density lipoprotein (LDL) receptor-related protein (LRP-1) was determined using OxiSelect diagnostic kits (Cell Biolabs, Inc., San Diego, CA, USA).

#### 2.4.4. Histological Examinations of the Brain

The histopathological examination covered the structure of the cerebral cortex, which performs separate, important vital functions in certain zones. Samples of the brain were cut in two lengthwise and fixed for 24 h in 5% formalin, pH = 7.2. Within 24 h, the fixed tissue fragments were passed through increasing concentrations of alcohol solutions, acetone, and xylene into paraffin blocks in a tissue processor (Leica TP-20). Paraffin-embedded microscope sections 5 μm thick were stained with haematoxylin and eosin (HE staining). Histological evaluation of the brain was carried out using a computer-assisted microscopic image analysis system. The system includes a light microscope (Nikon Eclipse E600, Melville, NY, USA) with a digital camera (Nikon DS-Fi1, Melville, NY, USA) and a PC with image-analysis software (NIS-Elements BR-2.20, Laboratory Imaging, Melville, NY, USA).

### 2.5. Statistical Analysis

The results are expressed as means and pooled SEM. Two-way analysis of variance (ANOVA) was used to determine the effect of the Cr source (Cr: none, Cr-Pic, Cr-Met and Cr-NPs) and the diet type (D: standard or high-fat low-fibre diet), and the interaction between these two factors (Cr × D). If the analysis revealed a significant interaction (*p* ≤ 0.05), the differences between treatment groups were then determined with Duncan’s post hoc test at *p* ≤ 0.05. The data were checked for normality prior to the statistical analyses. The statistical analysis was performed using STATISTICA software, version 10.0 (StatSoft Corp., Krakow, Poland).

## 3. Results

### 3.1. The Effect of a High-Fat Diet in Rats

The initial body weight of the rats used in the experiment was similar in the control and experimental groups and was about 130 g. In rats fed high-fat diet, the higher final body weight (*p* = 0.008) and lower daily feed intake (*p* = 0.042) compared to the control group were noticed. Administration of a high-fat diet to rats increased global DNA methylation in the brain and heart (*p* = 0.013 and *p* = 0.045, respectively) as well as MDA content (*p* = 0.048 and *p* < 0.001, respectively) compared to the group receiving a standard diet (Table 2). In addition, higher levels of 8-OHdG (*p* = 0.027) and MDA (*p* = 0.006) were noted in the blood of rats fed a high-fat diet than in those receiving a standard diet (Table 3). APE-1 (*p* = 0.029) and ANPG (*p* = 0.044) activity in the blood decreased in rats fed a high-fat diet (Table 4).

### 3.2. The Effect of Different Forms of Cr in the Diet of Rats

Of the three forms of Cr used in the diet, only the addition of Cr-NPs increased the final body weight (*p* = 0.048) of the rats compared to the control group. There was no effect of Cr addition to the rats’ diet regardless of the form on the daily feed intake. Compared to the group without a Cr supplement, the addition of Cr-NPs and Cr-Pic to the diet of rats increased the content of 8-OHdG in the heart (*p* = 0.047) and brain (*p* = 0.034), with the highest level noted in the group receiving Cr-NPs. Compared to the group without a Cr supplement, the addition of this element to the diet of rats, irrespective of its form, increased the level of MDA (*p* = 0.014) in the heart, reaching its highest value in the group receiving Cr-NPs. In the brain, however, the MDA level (*p* < 0.001) increased only in the group receiving Cr-NPs. The addition of Cr-Met and Cr-NPs to the diet of rats resulted in an increase in global DNA methylation (*p* = 0.009) in the brain relative to the group without Cr supplementation, with the highest percentage of methylated DNA observed in the brain of rats from the Cr-NPs group (Table 2).

The addition of Cr-NPs to the rat diet increased the blood PC content (*p* = 0.038) relative to the group without Cr. The inclusion of Cr-Pic and Cr-Met in the diet increased blood levels of 8-OHdG (*p* = 0.044), with the highest value of this parameter recorded in the group receiving Cr-Pic. MDA content (*p* = 0.029) in the blood decreased due to the addition of Cr-Pic, but increased in the rats receiving Cr-NPs compared to the group without Cr supplementation. The diet supplemented with Cr-Met and Cr-NPs decreased blood beta-amyloid levels compared to the group without a Cr supplement (Table 3).

Supplementation of the diet of rats with Cr-Pic increased TDG activity (*p* < 0.001) in the blood relative to the group without a Cr supplement, while supplementation with Cr-Met reduced the activity of this enzyme. APE-1 activity (*p* < 0.001) was decreased by Cr supplementation irrespective of the form used, reaching its lowest values in the groups receiving Cr-Met and Cr-NPs. The addition of Cr, irrespective of the form used, also decreased ANPG activity (*p* = 0.002) in the blood relative to the group without Cr supplementation, with the most pronounced reduction in the groups receiving Cr-Pic and Cr-Met. The inclusion of Cr-Pic and Cr-Met in the diet of rats decreased LPR-1 activity in the blood (*p* = 0.007). The addition of Cr-Met and Cr-NPs to the diet increased SOD activity (*p* < 0.001) compared to the group without added Cr, with the highest value observed in the case of Cr-NPs. An increase in GPx activity (*p* = 0.049) in the blood relative to the control group was noted in rats receiving Cr-NPs. The addition of Cr-Pic to the diet of rats resulted in a decrease in CAT activity (*p* = 0.033) in the blood compared to the group without Cr supplementation (Table 4).

### 3.3. Histological Examination of the Brain

**Figure 1:**
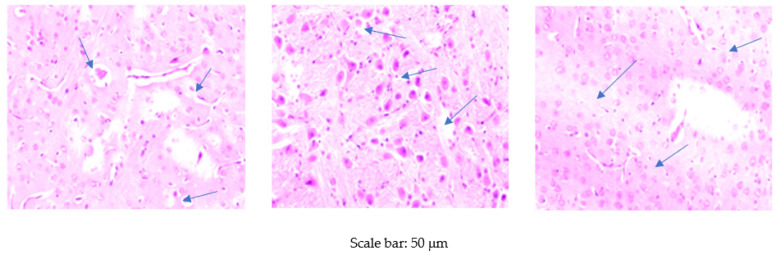
Morphological effects of high-fat diet and different Cr sources on rat brain. Foci of vacuolar degeneration of varying severity observed in all experimental groups, except the group receiving the standard diet without Cr supplementation (magnification 100×).

Foci of vacuolar degeneration of varying severity were observed in all experimental groups, except the group receiving the standard diet without Cr supplementation. However, they did not show a clear trend associated with the experimental group (Figure 1).

## 4. Discussion

Numerous scientific studies demonstrate that the use of a high-fat diet (especially long-term) impairs antioxidant defence mechanisms and leads to oxidative stress in the body [7,8]. Excessive fat intake results in mitochondrial β-oxidation of free fatty acids and excessive electron flow involving cytochrome c oxidase. This leads to overproduction of ROS, which damages proteins, lipids, and DNA, thereby disturbing the functions of certain cells [25]. A high-fat diet causes fat deposition in the body [7]. It seems that lipids are then the most susceptible to oxidation caused by increased ROS production. Our study showed that feeding rats a high-fat diet for eight weeks enhances blood lipid peroxidation processes, as evidenced by the increased content of MDA. Elevated blood MDA levels in rats due to a HF diet are also reported by Wu et al. [8], Meng et al. [26], and Khairunnuur et al. [27]. The results of our study also showed an increase in MDA levels in the brain and heart of rats receiving a high-fat diet. Increased lipid peroxidation manifested as increased MDA content in the heart of rats fed a high-fat diet, also reported by Noeman et al. [28]; in turn, in the brain by Maciejczyk et al. [29]. The brain, due to its high oxygen consumption, limited detoxification mechanisms, and substantial content of pro-oxidative metal ions, including Fe and Cu, is also highly susceptible to oxidative stress [30]. There are reports that the MDA produced at that time has mutagenic and carcinogenic properties, which disrupt proliferation of neurons, leading to their apoptosis. Furthermore, the presence of MDA in the brain increases the permeability of the blood-brain barrier, which may result in neurodegenerative changes in this organ [29,31].

In addition, our research showed that irrespective of its form, the addition of Cr to the diet of rats increased the level of MDA in the plasma, brain, and heart, with the most pronounced effect observed in the case of supplementation with Cr-NPs. This is consistent with results reported by Fatima et al. [32], who also found an increase in MDA content in the brain of rats receiving 50 µg/100 g BW or 200 µg/100 g bw Cr_2_O_3_NPs. This suggests that the addition of chromium to the diet, especially in the form of nanoparticles, adversely affects the body by inducing lipid peroxidation. Interestingly, the results of our research indicate that the addition of Cr-NPs to the diet negatively affects oxidation not only of lipids but of proteins as well. This is evidenced by the increased content of PC in the blood of the rats. An increased PC level is observed in many metabolic and neurodegenerative diseases— oxidative stress plays an important role in their pathogenesis [33]. The higher biological activity of Cr-NPs observed in our research compared to the other forms of Cr used in the experiment is probably linked to their ultra-small size, owing to which they are better absorbed in the digestive tract [34,35]. Moreover, there are reports that nanoparticles introduced into the body easily undergo Haber-Weiss and Fenton reactions, leading to increased ROS synthesis inducing oxidative stress, and thus to oxidation of cellular macromolecules [36]. However, the results of our research indicate that the addition of chromium to the diet in the form of a complex with methionine and as nanoparticles reduced the level of beta-amyloid in the blood of rats. Beta-amyloid is a neurotoxic peptide resulting from the proteolytic cleavage of amyloid precursor protein (APP) catalysed by beta-and gamma-secretase. It plays an important role in the pathogenesis of Alzheimer’s disease [37]. The available literature indicates that increased content of beta-amyloid formed in the body is closely correlated with intensifying oxidative stress [37,38]. However, the addition of dietary chromium may stimulate insulin activity, then in turn may result in the depletion of beta-amyloid outside the body [13]. Although insulin activity was not analyzed in this study, it may be assumed that this mechanism was partly responsible for the obtained results.

The increased production of ROS generated by a high-fat diet may result in oxidation not only of lipids but of nucleic acids as well [39]. Extensive oxidative damage interferes with replication and transcription, causing genetic instability and an increased number of mutations [40]. Assessment of the severity of oxidative damage to DNA commonly involves determination of the content of 8-hydroxysdeoxyguanosine (8-OHdG), an oxidized form of guanine [41]. Our research showed that feeding rats a high-fat diet increased the plasma level of 8-OHdG, which is consistent with results reported by Maciejczyk et al. [29]. Our results, however, did not show an increase in 8-OHdG levels in the brain or heart of rats receiving the HF diet. It can therefore be assumed that in both the brain and the heart, lipids are the first to undergo oxidation, as evidenced by the increase in MDA levels. Moreover, our experiment was relatively short, lasting eight weeks. It cannot therefore be ruled out that extending the experimental period would also increase oxidation of nucleic acids in the brain and heart of rats. Our research also showed that the addition of Cr to the diet of rats adversely affects DNA by increasing the content of 8-OHdG in the blood, heart, and brain. It should be noted, however, that Cr-NPs particularly enhance DNA oxidation in the organs, and Cr-Pic in the blood plasma. Hence, the results suggest that Cr-NPs have the highest genotoxicity among the tested chromium forms. Although Cr-Pic increased the plasma content of 8-OHdG, its composition may undergo rapid changes, e.g., due to filtration through the kidneys. Therefore, determination of 8-OHdG content in organs with relatively minor fluctuations in composition seems more reliable.

The available literature shows that a high-fat diet, in addition to enhancing DNA oxidation, can cause epigenetic changes in the body, of which DNA methylation is the best known [42,43]. In this process, methyl groups derived from S-adenosyl-L-methionine (SAM) attach to CpG islands containing cytosine residues [44,45]. As a consequence, gene expression is silenced with no change in the nucleotide sequence [43]. Our results showed that feeding rats a HF diet increases global DNA methylation levels in the brain and heart of rats. This is in conflict with results obtained by Ciccarone et al. [43], who recorded a reduction in DNA methylation in the heart of mice fed a high-fat diet. A decrease in the level of DNA methylation as a result of a HF diet can be assumed to be due to a reduced supply of key substances for this process, including methionine, which is necessary for SAM synthesis. However, it is very difficult to interpret the increase in global DNA methylation observed in the brain and heart in our study and to determine the mechanisms responsible for it. The addition of Cr to the diet of rats also adversely affected the genome by increasing levels of global DNA methylation in the brain, an effect that was most pronounced in rats receiving Cr-NPs. This can be assumed to be caused by the high biological activity of Cr nanoparticles. Excessive or abnormal attachment of methyl groups to CpG islands of important genes silences their expression. Consequently, numerous metabolic pathways may be disturbed as a result of a deficiency or complete lack of the proteins encoded by these genes [46].

As nucleic acids are relatively easily damaged by ROS, cells are equipped with complicated DNA repair mechanisms. DNA in cells can be repaired through a variety of mechanisms, but in the case of oxidative damage, base excision repair (BER) seems to play the most important role [47,48]. This process is determined by the activity of DNA glycosylases, which recognize the abnormal base and excise it. This enzyme family includes at least 11 different glycosylases, including apurinic/apyrimidinic endonuclease 1 (APE-1) and DNA-3-methyladenine glycosylase (ANPG) [47]. In our research, the use of a high-fat diet decreased both APE-1 and ANPG activity in the blood of rats. In contrast, the available literature indicates that in the case of increased ROS synthesis leading to DNA damage, the activity of repair enzymes, especially APE-1, increases [49]. Ding et al. [50], however, report that in the case of a significant increase in DNA damage in cells, a proportional reduction in repair enzyme activity can be observed. This is probably due to depletion of the enzymatic activity of the protein, which in conditions of constant mobilization cannot be effectively and quickly restored. Furthermore, there have been reports that an unbalanced high-fat diet can interfere with epigenetic regulation of DNA repair genes by changing the DNA methylation pattern. This may result in their abnormal expression, leading to reduced activity of the enzymes they encode [51]. The available literature indicates that the activity of DNA repair enzymes increases under the influence of oxidative stress, which intensifies the oxidation of nitrogen bases [49]. This is consistent with the increase in TDG activity observed in our study in rats receiving Cr-Pic, which generated excessive synthesis of 8-OHdG. In general, however, the addition of Cr to the diet of rats, irrespective of its form, decreased the activity of repair enzymes ANPG and APE-1. However, this effect can be assumed to result from depletion of repair enzyme activity due to excessive DNA damage.

Our research also showed a decrease in the activity of LRP-1 (low-density lipoprotein (LDL) receptor-related protein) in the blood of rats receiving Cr-Pic and Cr-Met supplements in their diet. This protein plays a key role in lipoprotein metabolism, protease degradation, and activation of lysosomal enzymes. LRP1 is also believed to take part in signalling pathways by interacting with other cellular receptors, including PDGFR-β and integrins. Thus, by modulating the response of smooth muscle cells to growth factors, it protects the vascular system. In addition, by influencing integrin functions, it modulates the integrity of the blood-brain barrier (BBB) [52]. In view of the above, the reduction in LRP-1 activity resulting from the addition of Cr-Pic and Cr-Met to the diet of rats should be considered an adverse effect.

The negative effect of the addition of Cr-NPs to the diet of rats on redox status was also confirmed by the assessment of the activity of key antioxidant enzymes. The strong increase in plasma SOD and GPx activity in rats receiving Cr-NPs, supported by an increase in the content of MDA, 8-OHdG and PC in the body, suggests that chromium induces severe oxidative stress and weakens the body’s antioxidant defence. Interestingly, the addition of Cr-Pic to the diet of rats caused a decrease in CAT activity in the blood of rats relative to the group without Cr supplementation. Since the addition of Cr-Pic increased plasma levels of MDA and 8-OHdG, the decrease in CAT activity can be assumed to be due to overproduction of ROS, which the enzyme was unable to effectively remove.

The histopathological examinations in our study showed foci of vacuolar degeneration in both the brain of rats fed the HF diet. The results are consistent with those reported by other authors [53,54]. He et al. [53] administered a high-fat diet to rats and found pathological changes in the endothelial cells of the cerebral vascular lumen, including nuclear swelling and an increase in the number of vacuoles. The results of both our own research and that of other authors confirm the harmful effects of a high-fat diet on the brain. The histopathological examination also showed adverse effects of Cr on the brain. The addition of Cr to the diet, irrespective of the form used, resulted in the development of foci of vacuolar degeneration in the brain. Similarly, Fatima et al. [32] noted the presence of small vacuoles and increased inflammation in the brain of rats receiving 50 µg/100 g BW of Cr_2_O_3_NPs for 14 days. Increasing the dosage of Cr_2_O_3_NPs to 200 µg/100 g BW caused cerebral necrosis and fibrosis manifested by pronounced lymphocytic infiltrates, cyst formation, neuronal vacuolization, and degeneration of the parenchyma. According to these researchers, adverse histological changes in the brain are due to excessive synthesis of ROS induced by Cr_2_O_3_NPs. However, this mechanism can be assumed to apply not only to Cr-NPs but to other forms of Cr as well.

## 5. Conclusions

The use of a high-fat diet intensifies epigenetic changes and oxidation reactions in the heart and brain of rats. It was concluded that it is not possible to limit the epigenetic changes and lipid, protein, and DNA oxidation in the heart and brain tissue of rats resulting from the use of a high-fat diet by supplementing the diet with chromium. The use of chromium in a high-fat diet was found to intensify adverse epigenetic and oxidative changes in the heart and brain of rats, especially in the case of supplementation in the form of Cr-NPs.

## Figures and Tables

**Table 1:** Composition of diets fed to rats, %.

Ingredient/Group	S	F
Casein ^a^	20.0	20.0
DL-methionine	0.3	0.3
Celullose ^b^	5.0	3.00
Sucrose	10.0	10.0
Rapeseed oil	8.0	8.0
Lard	-	17.0
Vitamin mixture ^c^	1.0	1.0
Mineral mixture ^d^	3.5	3.5
Choline chlorid ^e^	0.2	0.2
Cholesterol	0.3	0.3
Corn starch ^f^	51.7	36.7

^a^ Casein preparation (LACPOL Co., Murowana Goslina, Poland), crude protein 89.7%, crude fat 0.3%, ash 2.0%, and water 8.0%. ^b^ α-cellulose (SIGMA, Poznan, Poland), main source of dietary fibre. ^c^ AIN-93G-VM26, g/kg mix: 3.0 nicotinic acid, 1.6 Ca pantothenate, 0.7 pyridoxine-HCl, 0.6 thiamin-HCl, 0.6 riboflavin, 0.2 folic acid, 0.02 biotin, 2.5 vitamin B_12_ (cyanocobalamin, 0.1% in mannitol), 15.0 vitamin E (all-rac-α-tocopheryl acetate, 500 IU/g), 0.8 vitamin A (all-trans-retinyl palmitate, 500,000 IU/g), 0.25 vitamin D-3 (cholecalciferol, 400,000 IU/g), 0.075 vitamin K_1_ (phylloquinone), 974.655 powdered sucrose. ^d^ Mineral mix, g/kg mix: 357 calcium carbonate anhydrous CaCO_3_, 196 dipotassium phosphate K_2_HPO_4_, 70.78 potassium citrate C_6_H_5_K_3_O_7_, 74 sodium chloride NaCl, 46.6 potassium sulfate K_2_SO_4_, 24 magnesium oxide MgO, 18 microelement mixturee, starch to 1 kg = 213.62. ^e^ Microelement mixture, g/kg mix: 31 iron (III) citrate (16.7% Fe), 4.5 zinc carbonate ZnCO_3_ (56% Zn), 23.4 manganese (II) carbonate MnCO_3_ (44.4% Mn), copper carbonate CuCO_3_ (55.5% Cu), 0.04 potassium iodide KI, citric acid C_6_H_8_O_7_ to 100 g. ^f^ Corn starch preparation: crude protein 0.6%, crude fat 0.9%, ash 0.2%, total dietary fibre 0%, water 8.8%. Experimental sources of dietary Cr: chromium (III) picolinate (Cr-Pic), chromium (III)-methionine (Cr-Met), and nano-sized chromium (Cr-NPs) were added to the diet as an emulsion together with dietary rapeseed oil, not in the mineral mixture (MX). S—standard diet; F—high-fat, low-fibre diet.

**Table 2:** DNA methylation and indicators of oxidative changes in the heart and brain tissue of rats.

	Heart				Brain			
	DNA Methylation, %	PC *nmoL/mg Protein*	8-OHdG ng/g Tissue	MDA μmoL/kg Tissue	DNA Methylation, %	PC nmoL/mg Protein	8-OHdG ng/g Tissue	MDA μmoL/kg Tissue
Diet (D)								
S	4.985 ± 0.052 ^b^	1.013 ± 0.012	1.919 ± 0.047	4.112 ± 0.046 ^b^	8.164 ± 0.068 ^b^	1.606 ± 0.059	1.727 ± 0.061	5.501 ± 0.049 ^b^
F	5.123 ± 0.051 ^a^	1.083 ± 0.013	1.970 ± 0.048	4.384 ± 0.052 ^a^	8.796 ± 0.063 ^a^	1.609 ± 0.063	1.792 ± 0.057	8.183 ± 0.058 ^a^
Cr source (Cr)								
Without	5.0085 ± 0.056	0.9825 ± 0.007	1.867 ± 0.036 ^b^	4.091 ± 0.055 ^b^	8.329 ± 0.071 ^b^	1.569 ± 0.059	1.661 ± 0.067 ^b^	6.780 ± 0.057 ^b^
Cr-Pic	5.1245 ± 0.058	1.0785 ± 0.013	1.937 ± 0.041 ^a,b^	4.264 ± 0.049 ^a,b^	8.368 ± 0.069 ^b^	1.630 ± 0.062	1.838 ± 0.063 ^a^	6.845 ± 0.052 ^b^
Cr-Met	5.0155 ± 0.046	1.0825 ± 0.015	1.835 ± 0.031 ^b^	4.194 ± 0.052 ^a,b^	8.519 ± 0.068 ^a,b^	1.603 ± 0.058	1.678 ± 0.073 ^b^	6.677 ± 0.053 ^b^
Cr-NPs	5.0685 ± 0.051	1.0485 ± 0.014	2.138 ± 0.036 ^a^	4.443 ± 0.054 ± ^a^	8.705 ± 0.070 ^a^	1.629 ± 0.066	1.861 ± 0.059 ^a^	7.067 ± 0.059 ^a^
*p*-Value								
D effect	0.013	0.874	0.463	0.048	0.045	0.239	0.147	<0.001
Cr effect	0.158	0.269	0.047	0.014	0.009	0.057	0.034	<0.001
D × Cr interaction	0.158	0.587	0.355	0.478	0.884	0.043	0.034	0.962

Data were expressed as mean ± SD (for diet—S and F, n = 28; for Cr source—without, Cr-Pic, Cr-Met and Cr-NPs, n = 14). ^a,b^ Means within the same column differ significantly from the control group at *p* ≤ 0.05; PC—protein carbonyl, 8-OHdG-8-hydroxydeoxyguanosine, MDA—malondialdehyde; S—rats were fed a standard diet; F—rats were fed a high-fat, low-fibre diet; Cr-Pic—rats were fed diets (standard or high-fat, low-fibre) supplemented with 0.3 mg Cr/kg BW from chromium(III) picolinate; Cr-Met—rats were fed diets (standard or high-fat, low-fibre) supplemented with 0.3 mg Cr/kg BW from chromium (III)-methionine; Cr-NPs—rats were fed diets (standard or high-fat, low-fibre) supplemented with 0.3 mg Cr/kg BW from nano-sized chromium.

**Table 3:** DNA methylation, beta-amyloid content, and lipid, protein, and DNA oxidation indicators in rat blood.

	DNA Methylation, %	PC nmoL/mg	8-OHdG ng/mL	MDA µmol/mL	Beta-Amyloid pg/mL
Diet (D)					
S	23.65 ± 1.93	1.536 ± 0.023	9.871 ± 0.097 ^b^	1.113 ± 0.036 ^b^	6099.2 ± 27.46
F	25.04 ± 1.97	1.542 ± 0.021	11.07 ± 0.094 ^a^	2.405 ± 0.042 ^a^	5949.7 ± 28.92
Cr source (Cr)					
Without	23.93 ± 1.81	1.260 ± 0.017 ^b^	9.767 ± 0.087 ^b^	1.754 ± 0.046 ^a,b^	6689.6 ± 31.76 ^a^
Cr-Pic	24.45 ± 1.90	1.382 ± 0.0122 ^b^	11.53 ± 1.008 ^a^	1.525 ± 0.041 ^b^	6425.2 ± 29.64 ^a^
Cr-Met	25.22 ± 1.87	1.376 ± 0.022 ^b^	10.60 ± 0.095 ^a,b^	1.774 ± 0.051 ^b^	5540.6 ± 27.57 ^b^
Cr-NPs	23.78 ± 1.73	2.138 ± 0.019 ^a^	9.985 ± 0.085 ^b^	1.983 ± 0.057 ^a^	5442.6 ± 26.88 ^b^
*p*-Value					
D effect	0.064	0.369	0.027	0.006	0.078
Cr effect	0.564	0.038	0.044	0.029	0.007
D × Cr interaction	0.987	0.052	0.392	0.477	0.039

Data were expressed as mean ± SD (for diet—S and F, n = 28; for Cr source—without, Cr-Pic, Cr-Met and Cr-NPs, n = 14). ^a,b^ Means within the same column differ significantly from the control group at *p* ≤ 0.05; PC—protein carbonyl, 8-OHdG-8-hydroxydeoxyguanosine, MDA—malondialdehyde; S—rats were fed a standard diet; F—rats were fed a high-fat, low-fibre diet; Cr-Pic—rats were fed diets (standard or high-fat, low-fibre) supplemented with 0.3 mg Cr/kg BW from chromium(III) picolinate; Cr-Met—rats were fed diets (standard or high-fat, low-fibre) supplemented with 0.3 mg Cr/kg BW from chromium(III)-methionine; Cr-NPs—rats were fed diets (standard or high-fat, low-fibre) supplemented with 0.3 mg Cr/kg BW from nano-sized chromium.

**Table 4:** Activity of DNA repair and antioxidant enzymes in rat blood.

	TDG ng/mL	APE-1 ng/mL	ANPG ng/mL	LRP-1 ng/mL	SOD U/g Hb	GPx U/g Hb	CAT U/g Hb
Diet (D)							
S	12.96 ± 0.077	23.66 ± 0.099 ^a^	0.476 ± 0.009 ^b^	0.080 ± 0.005	49.39 ± 3.65	1939.8 ± 37.75	1886.9 ± 38.58
F	12.49 ± 0.072	19.13 ± 0.089 ^b^	0.589 ± 0.008 ^a^	0.079 ± 0.004	57.79 ± 3.86	2015.4 ± 39.65	1995.6 ± 35.97
Cr source (Cr)							
Without	13.92 ± 0.078 ^b^	26.04 ± 1.061 ^a^	0.602 ± 0.007 ^a^	0.084 ± 0.005 ^a^	48.03 ± 4.01 ^c^	1902.3 ± 35.62 ^b^	1972.5 ± 32.63 ^a,b^
Cr-Pic	14.53 ± 0.074 ^a^	24.91 ± 1.024 ^b^	0.492 ± 0.009 ^b^	0.075 ± 0.003 ^b^	48.92 ± 3.78 ^c^	1929.5 ± 35,93 ^b^	1842.8 ± 34.58 ^b^
Cr-Met	9.304 ± 0.077 ^c^	15.81 ± 0.092 ^c^	0.507 ± 0.008 ^b^	0.077 ± 0.006 ^b^	57.05 ± 3.99 ^b^	1954.1 ± 37.765 ^b^	1908.8 ± 38.92 ^a,b^
Cr-NPs	13.13 ± 0.069 ^b^	18.82 ± 0.094 ^c^	0.529 ± 0.008 ^a,b^	0.083 ± 0.006 ^a^	60.36 ± 4.21 ^a^	2124.6 ± 39.68 ^a^	2041.0 ± 39.48 ^a^
*p*-Value							
D effect	0.124	0.029	0.044	0.369	0.067	0.097	0.758
Cr effect	<0.001	<0.001	0.002	0.007	<0.001	0.049	0.033
D × Cr interaction	0.028	0.019	0.052	0.067	0.092	0.074	0.064

Data were expressed as mean ± SD (for diet—S and F, n = 28; for Cr source—without, Cr-Pic, Cr-Met and Cr-NPs, n = 14). ^a–c^ Means within the same column differ significantly from the control group at *p* ≤ 0.05; TDG—thymine DNA glycosylase, APE-1—apurinic/apyrimidinic endonuclease 1, ANPG—DNA-3-methyladenine glycosylase, LRP-1—low-density lipoprotein (LDL) receptor-related protein, SOD—superoxide dismutase, GPx—glutathione peroxidase, CAT—catalase; S—rats were fed a standard diet; F—rats were fed a high-fat, low-fibre diet; Cr-Pic—rats were fed diets (standard or high-fat, low-fibre) supplemented with 0.3 mg Cr/kg BW from chromium (III) picolinate; Cr-Met—rats were fed diets (standard or high-fat, low-fibre) supplemented with 0.3 mg Cr/kg BW from chromium (III)-methionine; Cr-NPs—rats were fed diets (standard or high-fat, low-fibre) supplemented with 0.3 mg Cr/kg BW from nano-sized chromium.
